# Characterization of the complete chloroplast genome of *Paphiopedilum micranthum*, an Endangered orchid in China

**DOI:** 10.1080/23802359.2019.1698330

**Published:** 2019-12-12

**Authors:** Xian-De Chen, Dong-Hui Peng, Si-Ren Lan, Jun Chen, Wen-Qun Fu

**Affiliations:** a College of Biological Science and Biotechnology, Minnan Normal University, Zhangzhou, China;; b Fujian Ornamental Plant Germplasm Resources Innovation and Engineering Application Research Center, Fuzhou, China;; c Key Laboratory of National Forestry and Grassland Administration for Orchid Conservation and Utilization at College of Landscape Architecture, Fujian Agriculture and Forestry University, Fuzhou, China

**Keywords:** *Paphiopedilum micranthum*, chloroplast genome, phylogenetic

## Abstract

*Paphiopedilum micranthum* is a rare species of terrestrial herb in the Orchidaceae. It is naturally distributed in southwestern China and north of Vietnam. Here, we reported the first complete chloroplast genome (cpDNA) of *P. micranthum*. The length is 163,243 bp, with 129 genes, including 77 protein coding genes, 38 tRNA genes, and 8 rRNA genes. It includes two inverted repeat regions (IRs) of 36,128 bp each, which were separated by a large single copy region (LSC) of 89,245 bp and a small single copy region (SSC) of 1742 bp. The overall GC-content of the whole chloroplast is 35.8%, while the corresponding values of the LSC, SSC, and IR regions are 33.2, 20, and 39.3, respectively. The complete chloroplast genome sequence of *P. micranthum* (GenBank accession number: MN535014) can be used as a useful resource for the evolutionary biology study of phylogenetic studies in Orchidaceae.

*Paphiopedilum micranthum* is a terrestrial or semi-epidemic plant of family Orchidaceae. It naturally distributed in southwestern Guangxi, south of Guizhou, southeastern of Yunnan in China and north of Vietnam (Zhongjian and Singchi 2009; Chen et al. 1999). Due to the high ornamental value and high cultivation value, it is one of the most popular orchid plants. However, *P. micranthum* is facing a sharp decline in its population due to anthropogenic effect. It has become Endangered because of loss of natural habitats and indiscriminate widespread collection. So far, no studies on the genome of *P. micranthum* have been published. Here, we determined the complete chloroplast genome sequence of *P. micranthum* (GenBank accession number: MN535014) to provide genetic and genomic information to promote its systematic research and conservation.

Fresh leaf sample of *P. micranthum* was acquired from Malipo County (N23°22′, E104°20′), Wenshan Prefecture, Yunnan Province of China and voucher specimen deposited at Herbarium of College of Forestry, Fujian Agriculture and Forestry University (specimen code 2010002). Total genomic DNA was extracted from fresh leaves using a modified CTAB method (Doyle and Dolyle 1987, Langmead and Salzberg 2012). Genome sequencing was performed using HiSeq2500 platform (Biodata Biotechnology Co. Ltd, Hefei, China). Genome sequences were screened out and assembled with Noveplastys (Huang and Madan 1999; Dierckxsens et al. 2016). The cp-genome was annotated with Geneious 11.0.5 (Kearse et al. 2012) and the map of the genome was generated using the program Organellar Genome DRAW (Lohse et al. 2013).

The complete chloroplast genome of *P. micranthum* is 163,243 base pairs (bp) in length. It exhibits a typical quadripartite structure of the large single-copy (LSC, 89,245 bp) and a small single-copy (SSC, 1,742 bp) regions, separated by a pair of inverted repeat regions (IRs, 36,128 bp each). The chloroplast genome contains a total of 129 unique genes constituting 77 protein-coding genes (PCG), 38 transfer RNAs(tRNA), and 8 ribosomal RNAs (rRNA). The overall GC-content of the whole chloroplast is 35.8%, while the corresponding values of the LSC, SSC, and IR regions are 33.2, 20. and 39.3%, respectively. To identify the phylogenetic position of *P. micranthum*, phylogenetic analysis was conducted. The maximum-likelihood (ML) phylogenetic tree was generated using species within the family Orchidaceae by MEGA 7.0 (Kumar et al. 2016), the branch support was computed with 1000 bootstrap. The results indicated that *P. micranthum* was closely related to *Paphiopedilum delenatii*. *Paphiopedilum micranthum* and the other 15 species were clustered into a clade. This unique sequence divergence identified in the cp DNA will serve as a molecular basis for identification of this species as well as phylogenetic study of Orchidaceae (Figure 1). This unique sequence divergence identified in the cp DNA will serve as a molecular basis for identification of this species as well as phylogenetic study of Orchidaceae.

**Figure 1.**
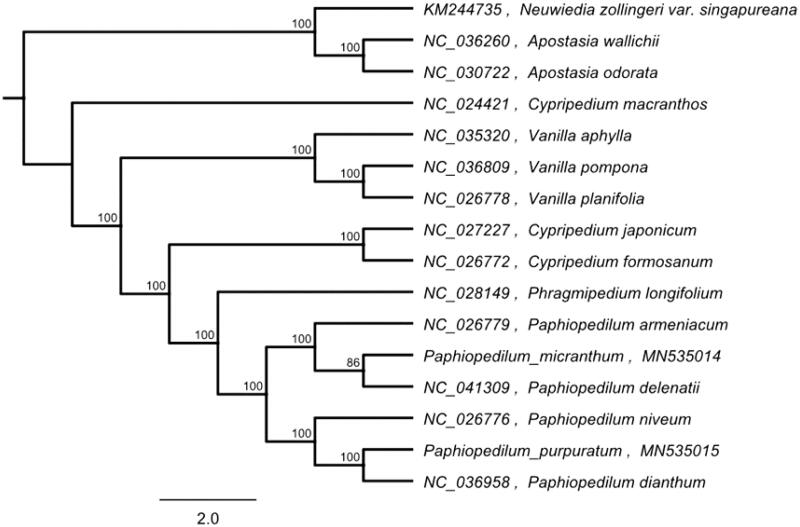
Maximum likelihood tree based on the complete cp genome sequences of 16 species from the Orchidaceae. Shown next to the nodes are bootstrap support values based on 1000 replicates.
